# A model combining deep learning and ensemble learning for melanoma recognition via dermoscopy

**DOI:** 10.3389/fonc.2026.1782150

**Published:** 2026-05-15

**Authors:** Jinyan Jiang, Suqing Yang

**Affiliations:** Dermatology, Heilongjiang Zhongyiyaodaxue Fushu Dier Yiyuan, Harbin, Heilongjiang, China

**Keywords:** deep learning, dermoscopy, ensemble learning, melanoma, XGBoost

## Abstract

**Background:**

Accurate identification of melanoma is key to improving prognosis, while traditional dermatoscopic recognition suffers from subjectivity and poor consistency. This study aimed to develop a deep learning-ensemble model to enhance the performance of dermatoscopic differential diagnosis between malignant melanoma and other malignant skin lesions.

**Methods:**

Using the ISIC-2024 and HAM10000 dermatoscopic datasets, nine convolutional neural network models (DenseNet201, EfficientNetB7, EfficientNetV2S, InceptionResNetV2, MobileNetV3Large, NASNetLarge, ResNet50V2, VGG19, and Xception) were employed for initial classification; the raw scores from each model were then used as feature vectors and input into an XGBoost ensemble model to construct the final classification model. Model performance was validated through accuracy, precision, recall, F1 Score and area under the curve (AUC).

**Results:**

All nine models significantly distinguished between the two classes (malignant melanoma and other malignant skin lesions; all p<0.001), with AUCs on the test dataset ranging from 0.921-0.967; the XGBoost ensemble model achieved an AUC of 1.00 on the training dataset and 0.988 on the test dataset, with Xception as the primary feature source.

**Conclusion:**

The integration of deep learning and ensemble learning strategies can improve dermatoscopic melanomadifferential diagnosis accuracy, provide technical support for clinically assisted recognition and possessing potential for clinical translation.

## 1 Introduction

Melanoma, a highly malignant skin tumor, is experiencing a rapidly increasing incidence rate (1). Early and accurate detection is a key factor in improving patient prognosis and treatment success rates (2). In clinical practice, dermoscopy, due to its ability to clearly visualize the microscopic structure of skin lesions, has become a common method for melanoma screening (3). Meanwhile, the application of artificial intelligence (AI) technology in the field of skin lesion image recognition is being explored more deeply, providing new directions for improving recognition efficiency and accuracy (4). Although the overall incidence of melanoma is relatively low, its extremely high malignancy and the limited number of cases encountered by physicians in some regions, leading to insufficient clinical experience, indicating that accurate identification remains a pressing industry challenge (5).

Melanoma identification still faces many challenges that require breakthroughs. First, traditional dermoscopy relies heavily on physicians’ clinical experience, introducing a degree of subjectivity, especially in primary healthcare institutions or in situations where experience is limited, leading to missed diagnoses and misdiagnoses^5 (^6^),^. Second, existing AI identification models may suffer from training data bias, making it difficult to meet diverse clinical needs. For example, some deep learning-based identification models perform exceptionally well on specific datasets (7), but their accuracy is low when applied to dermoscopy images acquired from multiple centers and devices, making them unsuitable for real-world clinical procedures (8).

With the development of AI technology, significant progress has been made in the field of melanoma recognition, with breakthroughs in deep learning and radiomics technologies being the most prominent (9–11). Previous studies have investigated melanoma detection using single convolutional neural networks (CNNs) based classification models (12), radiomics and handcrafted feature-based approaches focusing on interpretability and generalizability (13, 14), as well as fusion and ensemble learning strategies including simple averaging, weighted fusion, and stacking or meta-learning frameworks (15–17). In deep learning, models represented by convolutional neural networks CNNs have demonstrated excellent performance in dermoscopic image classification tasks, with recognition accuracy often comparable to that of dermatologists. These models can efficiently complete core tasks such as lesion segmentation, feature extraction, and classification, improving the automation level of the recognition process (15, 16). Radiomics, as an emerging image analysis technology, has been explored in melanoma diagnosis and metastatic disease assessment scenarios through high-throughput feature extraction and quantitative analysis of medical images. Related models have achieved an area under the curve (AUC) of over 0.8, demonstrating strong discriminative efficacy (18). In recent years, the integrated application of these two technologies has become a research hotspot. Previous studies have constructed non-invasive recognition models combining deep learning and radiomics, improving the accuracy and portability of melanoma recognition by extracting deep and quantitative features from images (19).

Significant progress has been made in deep learning and radiomics technologies. However, research gaps remain. First, existing models focus primarily on combining single deep learning models with radiomics features, failing to fully explore the complementary features of multiple deep learning models, and research on the integration and optimization of multi-model features is insufficient (20, 21). Second, strategies for fusing radiomics features with deep learning features in dermoscopy images still need improvement (22, 23). Ensemble learning strategies, including XGBoost ensemble learning, have been proven to be effective methods for model integration (24). Third, existing models lack large-sample, multi-center external validation, making it difficult to support clinical decision integration (23, 25). Furthermore, there are few high accuracy AI models for population-based melanoma identification, making it difficult to solve the identification challenges in clinical diagnosis and treatment.

Distinguishing melanoma from other malignant skin lesions represents a critical clinical diagnostic challenge, as their treatment strategies and prognoses differ substantially. The model proposed in this study is designed to address this specific differential diagnosis and provide an artificial intelligence–assisted tool for precise classification of cutaneous malignancies. Specifically, multiple classic CNNs models were first used for preliminary classification of dermoscopic images. Then, the raw scores output by each model are used as ensemble features, and an XGBoost ensemble learning model is employed to complete the final classification decision. Simultaneously, the model’s generalization performance on external independent datasets is validated. This study has significant clinical and academic value. First, by employing a multi-model ensemble strategy, it improves the accuracy and stability of melanoma identification, providing an auxiliary identification tool for clinical practice, helping to reduce missed diagnoses and misdiagnoses, and supporting early and precise intervention. Second, by focusing on dermoscopic images as a core clinical modality and optimizing the fusion strategy of deep learning and ensemble learning, it provides methodological references for the clinical translation of related technologies. This study contributes to improving the accuracy and portability of melanoma identification, ultimately improving patient prognosis.

The main contributions of this study are as follows: (1) To address subjectivity and poor consistency in conventional dermatoscopic melanoma identification, this study developed a novel model integrating deep learning and ensemble learning. (2) Using two well-established dermatoscopic datasets, we evaluated nine CNN models and built an XGBoost ensemble model with their output scores as feature vectors. (3) This model achieves accurate melanoma identification, may provide technical support for clinically assisted recognition, and holds promising clinical translation potential.

## 2 Materials and methods

### 2.1 Data sources

This study used the ISIC-2024 dataset (https://challenge2024.isic-archive.com) of the International Skin Imaging Collaboration (ISIC) as the training dataset (26) and HAM10000 as the test dataset (https://www.kaggle.com/datasets/kmader/skin-cancer-mnist-ham10000) as the test dataset (27). The dataset contains dermoscopic image samples confirmed by clinical pathology. Benign samples and samples with missing diagnoses were removed, retaining only dermoscopic images of pathologically confirmed malignant melanoma and other malignant lesions. Finally, the samples were categorized into two classes, malignant melanoma (classified as Melanoma) and other skin cancers (classified as Malignant-other). The training dataset has a total sample size of 8846, including 7223 cases of Melanoma and 1623 cases of other skin cancers; the test dataset has a sample size of 1954, including 1113 cases of Melanoma and 841 cases of other skin cancers (**Supplementary Table 1**).

### 2.2 Data preprocessing

The image analysis workflow was designed to ensure standardized preprocessing and reproducible model training. All dermoscopic images underwent a uniform preprocessing pipeline prior to model development. Initially, images were resized and center-cropped to a fixed spatial resolution of 224 × 224 pixels to ensure dimensional consistency across samples. Images were subsequently converted to three-channel RGB format. The processed images were then transformed into numerical tensors suitable for model input. For the training dataset, random horizontal flipping was applied as a data augmentation strategy (trigger probability = 0.5) to enhance model robustness and mitigate overfitting.

### 2.3 Model construction and training

This study constructs a two-level model architecture of a “basic deep learning model + XGBoost ensemble learning decision”. First, the basic deep learning convolutional neural network CNNs completes the preliminary feature classification of dermoscopy images. Then, the Raw Score (Raw_Score) of each deep learning model is used as the feature vector and input into the XGBoost ensemble model to complete the final melanoma identification. The Raw Score refers to the output layer raw output scores generated by each base CNN model for all samples before sigmoid activation, while the sample prediction probability is derived from the sigmoid function during model evaluation. Compared with probability values compressed to the (0,1) interval by the sigmoid function, these unbounded raw scores retain complete prediction confidence information and avoid the loss of feature discrimination caused by gradient saturation in the high-confidence interval. Their characteristics are more compatible with the splitting logic of the XGBoost tree model, which can enhance the ensemble model’s ability to capture output differences across different CNNs, while may control overfitting risk and improving the model’s generalization performance.

When constructing and training the basic deep learning models, nine types of CNNs suitable for medical image classification were selected as the basic models: DenseNet201 (Densely Connected Convolutional Networks 201); EfficientNetB7 (Efficient Convolutional Neural Network B7); EfficientNetV2s (Efficient Convolutional Neural Network V2 small); InceptionResNetV2 (Inception-Residual Convolutional Neural Network V2); MobileNetV3Large (Mobile Convolutional Neural Network V3 Large); NASNetLarge (Neural Architecture Search Network Large); ResNet50V2 (Residual Neural Network 50 V2); VGG19 (Visual Geometry Group 19-layer Convolutional Neural Network); and Xception (Extreme Inception Convolutional Neural Network). All the models adopted the same training strategy and hyperparameters. These nine models are CNN architectures widely used in medical image classification tasks, selected as the base models in this study. These architectures cover multiple mainstream network design paradigms (including dense connection, residual, attention-optimized and lightweight architectures), to ensure comprehensive and discriminative feature extraction from complex dermoscopic images of different malignant skin lesions, thus providing input for subsequent ensemble learning to achieve differential diagnosis.

All base CNNs were initialized with pretrained weights to leverage transfer learning and improve convergence efficiency. For binary classification, the final fully connected layer was replaced with a single-neuron output layer. Model optimization was performed using the Adam optimizer with a weight decay coefficient (λ = 1 × 10^-4^) to mitigate overfitting through L2 regularization. The initial learning rate was set to 1 × 10–^4^ and adjusted using a cosine annealing schedule (T_max = 50), with a minimum learning rate of 1 × 10–^6^ to facilitate stable convergence. Weighted sampling strategies are employed, and class-weighted loss functions are used. Each model was trained for 30 epochs. During training, model outputs were passed through a sigmoid activation function to obtain probability estimates in the range [0,1]. A classification threshold of 0.5 was applied to determine the predicted class label (>0.5 is Malignant class, ≤0.5 is Malignant-other class). At each epoch, performance metrics including accuracy, precision, recall, F1-score, and AUC were computed. Model selection was based on the highest F1-score achieved during training, considering the imbalanced nature of the dataset. The optimal model weights were retained for subsequent evaluation. Following training, batch inference was conducted separately on the training and independent test datasets. Predicted probabilities and class labels were generated, and performance metrics were calculated to quantify classification effectiveness and generalization ability.

The XGBoost ensemble learning model uses the Raw Score output by 9 basic deep learning models as input features to complete the final melanoma identification decision. The construction and training process of the XGBoost ensemble learning model is as follows: (1) Feature extraction and standardization: Input the training dataset and test set images into the 9 basic deep learning models that have been trained, and extract the Raw Score of the two classes of samples output by each model as the ensemble feature vector. (2) XGBoost model initialization: Initialize the classification model based on the official XGBoost API. The core hyperparameters are fixed as follows: learning rate = 0.01, number of decision trees = 500, tree depth = 6, subsample ratio = 0.8, column sampling ratio = 0.9, and the loss function is class-weighted loss functions. (3) Integration model training: The standardized feature vectors and corresponding labels are input into the XGBoost model, and the model training is completed using 5-fold cross-validation. The AUC of the test dataset is used as the core evaluation index for hyperparameter optimization. After training, the optimal model weights are saved for subsequent model validation and evaluation. (4) Inference process: Input the dermoscopic images of the test dataset to be identified, input the Raw Score features of the 9 basic deep learning models, input them into the XGBoost integration model, and output the melanoma identification results. Finally, for the XGBoost integration model, the feature importance is calculated to quantify the feature contribution of the 9 basic deep learning models. The feature importance calculation adopts the gain method, that is, the proportion of the reduction of the total loss of each feature to the model, reflecting the influence of the single basic model on the integration decision. The contribution of each CNN model to the XGBoost integration result is visualized to identify the core effective features.

### 2.4 Model validation and evaluation

To evaluate the generalization ability of the model results, the independent test dataset HAM10000, which was not involved in the model training, is input into the final trained model, and the test set evaluation metrics are output as a performance reference for external model validation.

Classification performance and robustness of the model were quantified by calculating a set of metrics for both the training and test datasets, including: (1) Basic classification metrics: accuracy, precision, recall, and F1 score; (2) Receiver operating characteristic (ROC) curve and AUC, where AUC reflects the overall discrimination ability of the model; (3) Distribution feature metrics: raw scores (outputs of each base CNN model before sigmoid activation) were extracted, and the score distribution features of Melanoma and Malignant-other samples in both datasets were analyzed.

Statistical significance of the classification results was verified via independent samples t-test, examining the difference in Raw Score distribution between Melanoma and Malignant-other samples in the training and test datasets. The significance level was set at α=0.05 (two-tailed), with p<0.05 considered statistically significant. 95% confidence intervals (95% CI) were calculated for core metrics including AUC and F1-Score to quantify the uncertainty of evaluation results; a AUC ≥ 0.6 was defined as the threshold for classification value.

### 2.5 Software and experimental environment

All deep learning models were developed and trained using TensorFlow (version 2.15.0) with Keras (version 2.15.0) as the high-level neural network API. Ensemble learning was implemented using XGBoost (version 2.0.3) for both model training and inference. Data preprocessing and manipulation were conducted using NumPy (version 1.26.3) and Pandas (version 2.2.0), while scikit-learn (version 1.4.0) was employed for auxiliary machine learning utilities and data balancing procedures. Image preprocessing operations were performed using OpenCV (version 4.9.0) and Pillow (version 10.2.0). Statistical analyses and numerical computations were carried out using SciPy (version 1.12.0). Figures were plotted using Matplotlib (version 3.8.2).

All model training procedures were executed with GPU acceleration enabled via CUDA to enhance computational efficiency. The experiments were conducted using Python (version 3.12.1). Model training was performed on a system equipped with an NVIDIA RTX 3060 GPU with 12 GB of VRAM, utilizing CUDA version 12.6 and cuDNN version 8.9.7 for hardware acceleration. The operating system used was Windows 10 (build 19045.6456).

## 3 Results

### 3.1 Performance of basic deep learning models

**Figure 1:**
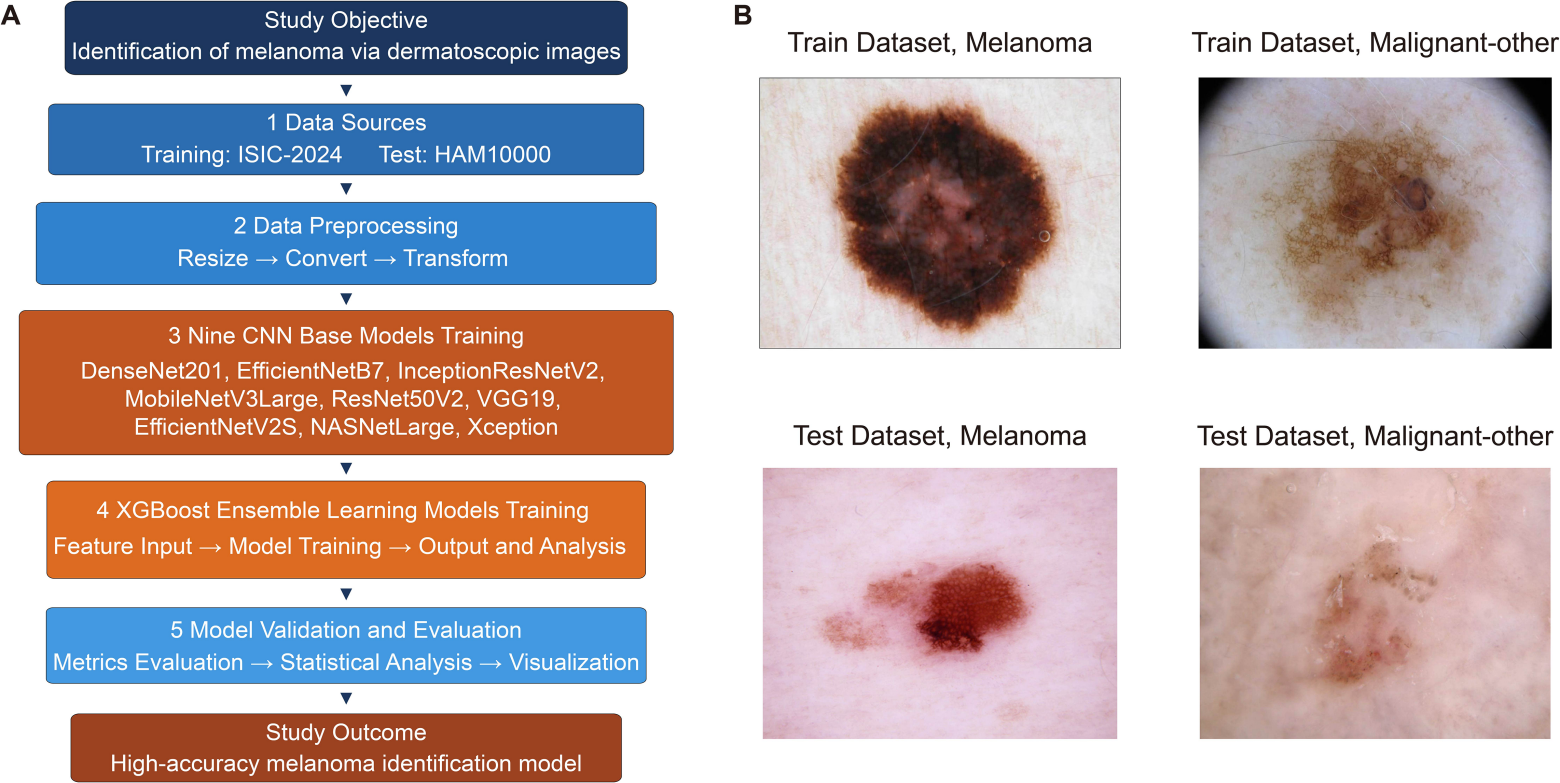
Technical workflow and representative case images. **(A)** technical flowchart of this study. **(B)** representative case images. AUC, area under the curve; Melanoma, malignant melanoma; Malignant-other, other skin cancers.

**Figure 2:**
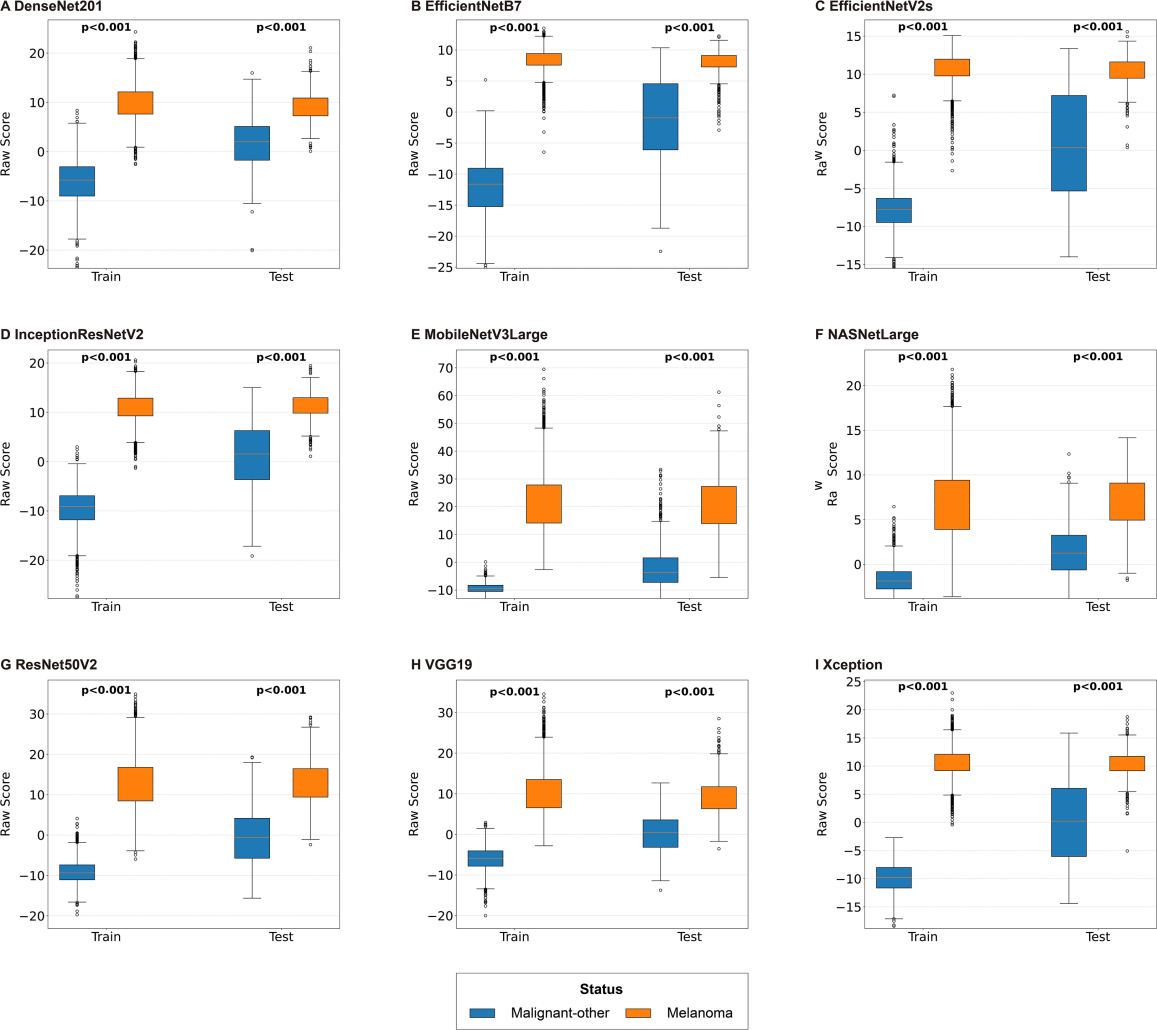
Distribution of raw scores for deep learning models. Each subplot represents the results of different single model. P < 0.001 indicates a statistical significance of the difference between groups; the vertical axis represents the raw score, and the horizontal axis represents the sample grouping (Train/Test).

The technical process of this study is shown in **Figure 1A**. Representative case images are shown in **Figure 1B**. Completing the training and evaluation of the basic deep learning models, the analysis of the raw score distribution revealed that there were significant differences in the raw score between Malignant-other and Melanoma in both the Train and Test groups of each model (all p<0.05), confirming that all models were statistically significant in distinguishing between the two types of samples in both the Train and Test groups (**Figure 2**; **Supplementary Table 2**).

**Figure 3:**
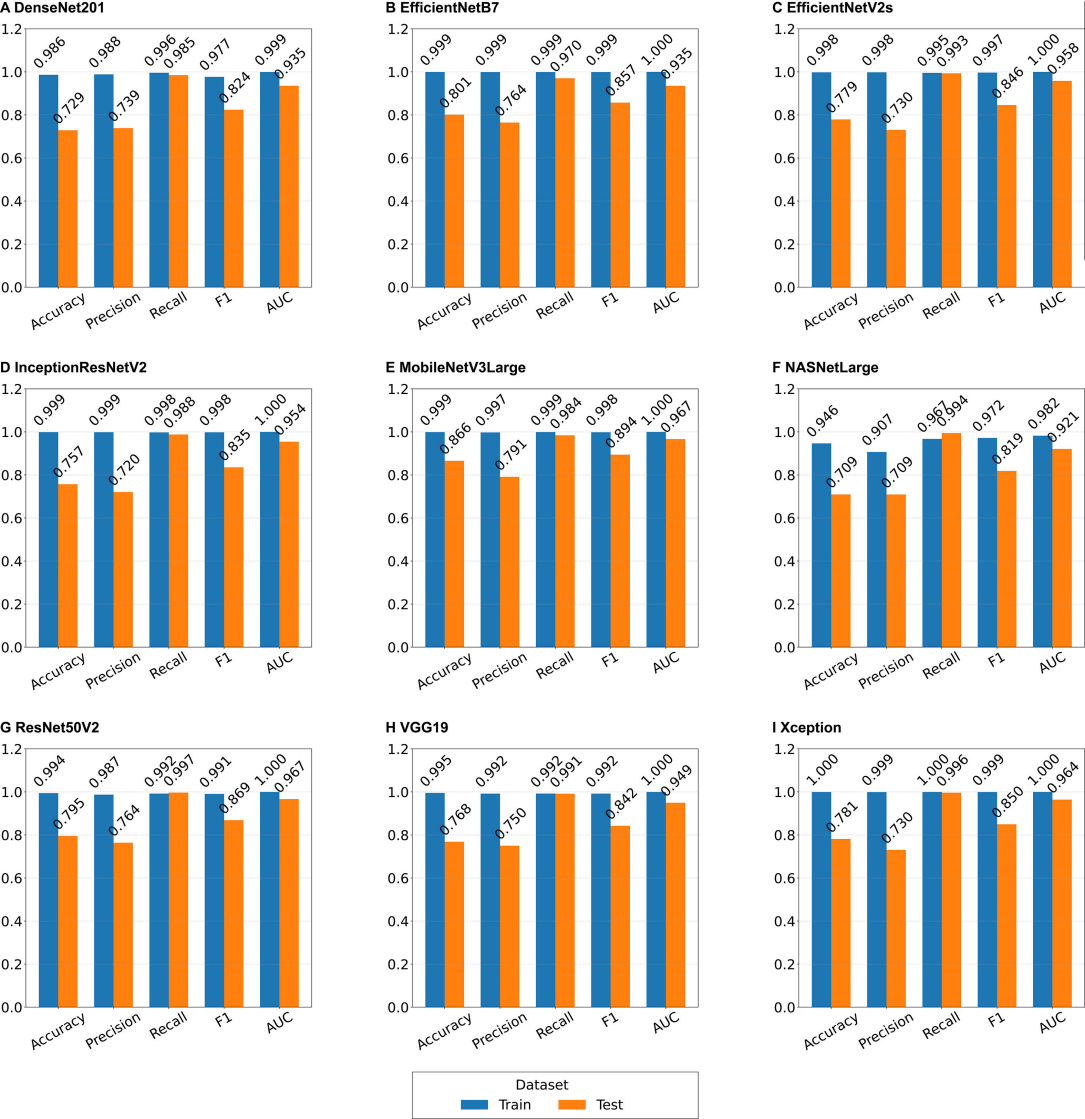
Performance of deep learning models. Each subplot represents the results of different single model. The vertical axis represents the indicator score (0-1); the horizontal axis represents the performance indicator; AUC, area under the curve.

Of nine deep learning models in the train and test groups revealed that Xception achieved near-perfect training performance (1.000 accuracy, 1.000 AUC) and robust test generalization (0.781 accuracy, 0.964 AUC). MobileNetV3Large and ResNet50V2 delivered the top test AUC (both 0.967), with MobileNetV3Large reaching the highest test accuracy (0.866) across all algorithms. EfficientNetB7, EfficientNetV2s, InceptionResNetV2 and VGG19 all reached 1.000 training AUC, with stable test AUC ranging from 0.935 to 0.958 and test recall consistently above 0.970. DenseNet201 maintained balanced performance, with 0.986 training accuracy and 0.955 test AUC. NASNetLarge showed the weakest overall performance, with the lowest training (0.946) and test (0.709) accuracy, and the minimum test AUC (0.921). All models exhibited good training fitting, but had limited test accuracy and precision, indicating existing sample misclassifications and optimization space (**Figure 3**; **Supplementary Table 3**).

**Figure 4:**
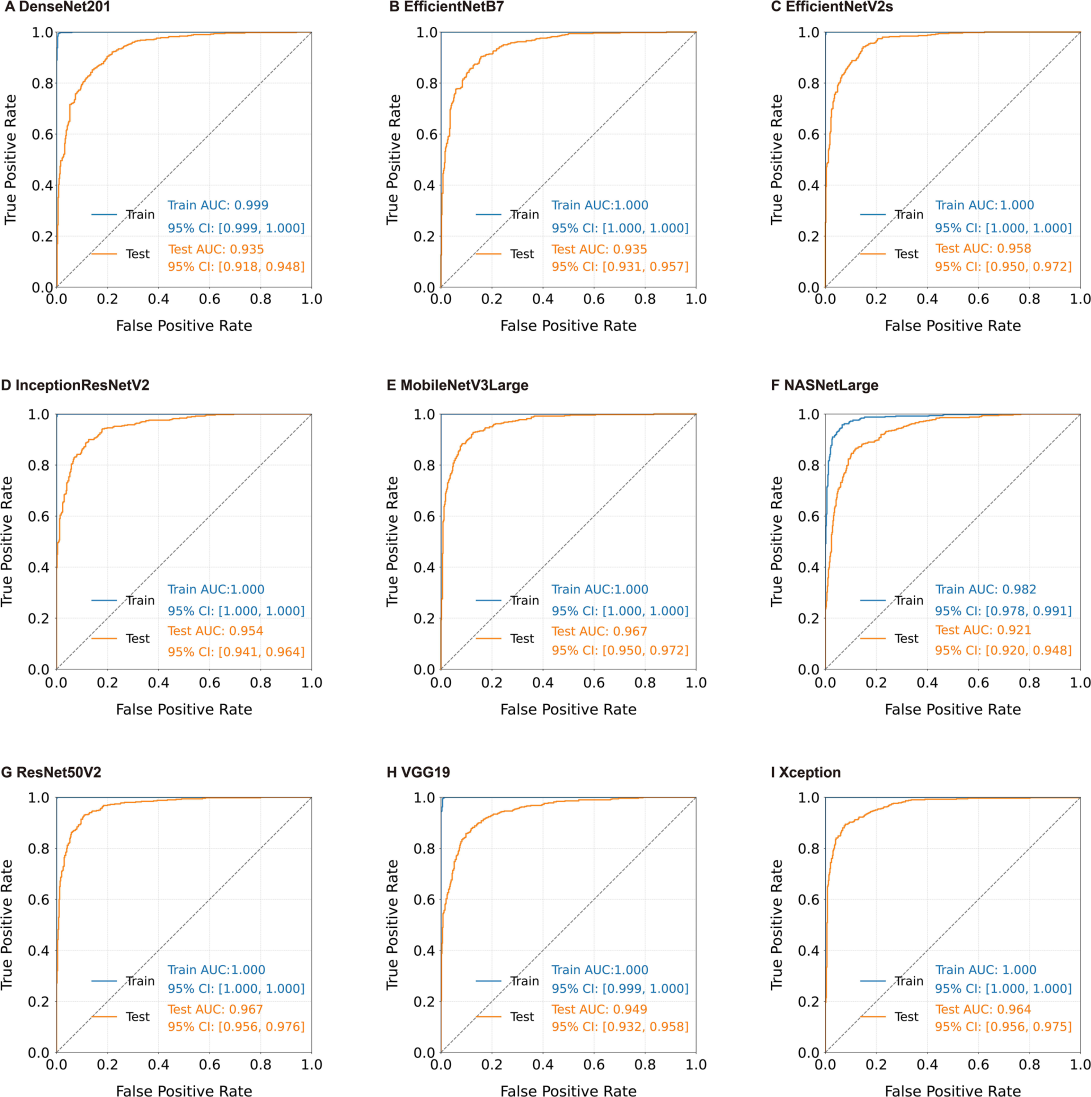
ROC curve of deep learning models. Each subplot represents the results of different single model. AUC, area under the curve; CI, confidence interval.

The F1 score and AUC results indicate that the model achieved good classification performance overall, but there is a risk of misclassifying malignant melanoma as non-malignant melanoma. Further analysis of the ROC curves of the nine deep learning models shows that, except for NASNetLarge, the AUCs of the other models in the Training group are close to or reach 1.000, and the AUCs in the test group are 0.935-0.967, demonstrating good predictive and discriminative abilities. NASNetLarge performs weakly, with an AUC of 0.982 in the training group and 0.921 in the test group (95% CI: 0.920, 0.948), possessing only some predictive and discriminative ability (**Figure 4**). MobileNetV3Large exhibits the best classification performance (AUC = 0.967; **Figure 3E**; **Figure 4E**).

### 3.2 Performance of ensemble learning models

**Figure 5:**
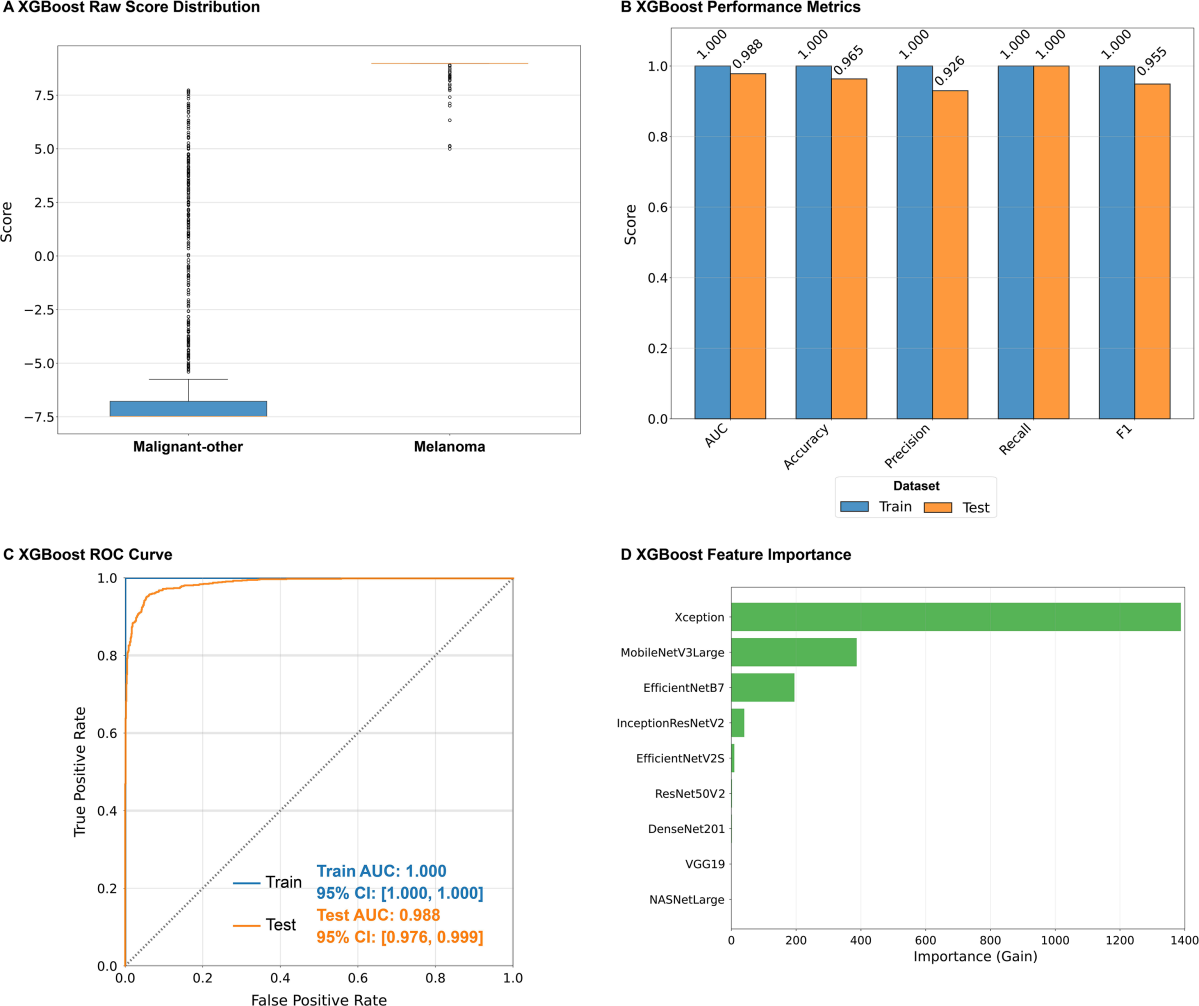
Performance and contribution of the ensemble model. **(A)** XGBoost Raw Score Distribution. **(B)** XGBoost Performance Metrics. **(C)** XGBoost ROC Curve. **(D)** XGBoost Feature Importance. AUC, area under the curve; CI, confidence interval; XGBoost, extreme gradient boosting.

The next step involved analyzing the XGBoost ensemble model. The results revealed that the raw score of the XGBoost ensemble model clearly distinguished between Malignant-other and Melanoma (p<0.001; **Figure 5A**; **Supplementary Table 4**). The train group achieved excellent performance with all performance metrics reaching 1.00, while the test group maintained a AUC of 0.988, with good accuracy and other metrics, demonstrating strong classification ability (**Figure 5B**; **Supplementary Table 5**). The ROC curves confirmed that both groups had excellent predictive and discriminative abilities (train dataset AUC = 1.00, test dataset AUC = 0.988; **Figure 5C**). The ensemble learning model revealed a performance improvement compared to the basic deep learning model (**Figures 3**, **5B**). Xception, MobileNetV3Large, and EfficientNetB7 were the three features that contributed the most to the ensemble learning model (**Figure 5D**).

## 4 Discussion

This study revealed that all nine basic deep learning models could distinguish between melanoma and malignant-other samples in dermoscopy images (all p < 0.001; **Figure 2**), and most models revealed good classification performance on the independent test dataset (AUC: 0.921-0.967; F1 score: 0.824-0.894), demonstrating good classification ability (**Figure 3**). ROC curve analysis of the deep learning models revealed that, except for NASNetLarge, the other models had good predictive discrimination ability (AUC ≥0.921) on the test dataset, with EfficientNetB7 and EfficientNetV2s achieving perfect prediction (**Figure 4**). The ensemble learning model based on XGBoost further improved the recognition reliability, with an AUC of 1.00 on the training dataset and an AUC of 0.988 on the test dataset. Feature importance analysis confirmed that the Xception model was the largest contributor of features in the ensemble system (**Figure 5**). In this study, the AUC (0.921) of NASNetLarge was lower than that of other models. It is speculated that this is because the network structure of NASNetLarge is more complex and its adaptability to small and medium-sized datasets such as dermoscopy images is insufficient, making it prone to underfitting. In contrast, the MobileNetV3Large model, due to its composite scaling strategy, achieves a better balance between parameter efficiency and feature extraction capability, thus realizing high-performance prediction (28).

Regarding the significant ability of deep learning models to distinguish dermoscopic melanomas, all nine models in this study were able to differentiate between the two classes of samples on both the training and test sets. This is consistent with the results of a dermoscopic melanoma identification study based on two waves of ISIC datasets, which confirmed that the AUC of deep learning models for classifying skin lesions can reach 0.96 (29). Combining the results of existing study and this study, deep learning models can effectively identify lesion features in dermoscopic images, which may align with the core observations made by clinicians.

One study constructed an artificial neural network for handcrafted expert features and compared and fused it with seven advanced convolutional neural networks; this was presented at ISIC. Experiments on a subset of the 2019 dataset (6296 nevi and 1361 melanomas) revealed that the AUC of the fused algorithm reached 0.942, outperforming single methods (22). Similarly, a study developed an automated system for classifying lesions and retrieving similar melanoma cases. The method proposed a deep learning framework based on transfer learning, using multiple pre-trained models to extract deep features, followed by weighted fusion and ensemble learning for classification. Performance was evaluated by accuracy and average precision. The results revealed that the ensemble model significantly outperformed independent models. These findings confirm the effectiveness of fusion methods, improving the accuracy and robustness of melanoma identification and potentially enhancing diagnostic efficiency in clinical practice. This study is the first to construct an XGBoost ensemble model after building a basic deep learning model (30). The ensemble model achieved an AUC of 1.00 on the training dataset and 0.964 on the test dataset, outperforming the single XGBoost skin lesion recognition model (31) and demonstrating that multi-model ensemble strategies can improve recognition stability by fusing the feature advantages of different models (32). The results clearly show that Xception is the core feature source, and the ensemble learning feature representation ability of this study is superior to a single CNN model by 20% (31). As reported in previous studies, single-model optimization can further enhance the performance of deep learning models in disease classification and prediction (33–35).

In summary, this study demonstrates that nine basic deep learning models can distinguish between melanoma and malignant-other samples in dermoscopic images. Most models exhibit good classification performance, and the XGBoost-based ensemble model further enhances recognition reliability, clearly identifying Xception as the core feature source. This study employs a two-step construction strategy of “basic deep learning model + XGBoost ensemble”, overcoming the limitations of single models. The ensemble model outperforms existing single XGBoost skin lesion recognition models, pinpointing the core feature source. The significance of this study lies in validating the effectiveness of deep learning and ensemble strategies in dermoscopic melanoma identification, providing auxiliary diagnostic evidence for clinical practice; it also clarifies the advantages and core feature sources of multi-model ensembles, providing a reference for related algorithm optimization. Furthermore, the results echo and expand upon existing fusion methods, promoting the clinical translation of deep learning technology and contributing to improved efficiency and accuracy in early melanoma diagnosis.

This study may have certain limitations. The data source is based mainly on publicly available datasets and is a retrospective study. The model does not incorporate multi-dimensional information such as clinical history and pathological features, resulting in a relatively singular recognition dimension. It should be noted that the majority of melanoma samples in the training dataset leads to sample imbalance, and the generalizability of our study’s results should be carefully validated in real-world clinical settings. Future research directions could focus on expanding the sample size to include dermoscopy image data from multiple centers to improve the clinical applicability of the model. It also needs to integrate radiomics features with clinical information to build a multimodal recognition model and promote its clinical translation.

## 5 Conclusion

Melanoma identification relies on dermoscopy but is easily influenced by physician experience, leading to a high risk of missed or misdiagnosis. This study aims to construct a model combining deep learning and ensemble learning to improve the accuracy of dermoscopic melanoma identification. Nine classic convolutional neural network models were selected for feature extraction and preliminary classification of dermoscopic images. The original scores of each model were used as feature vectors and input into an XGBoost ensemble model for final classification. The model performance was validated by stratified sampling of the dataset. The results revealed that all nine deep learning models could significantly distinguish melanoma from malignant-other samples, with most models achieving an AUC of 0.921-0.967 on the test dataset. The XGBoost ensemble model had an AUC of 1.00 on the training dataset and 0.988 on the test set, with Xception being the core feature source. These results improve the accuracy and stability of melanoma identification, providing an auxiliary identification tool for clinical practice. This study demonstrates the significant advantages of combining deep learning and ensemble learning in dermoscopic melanoma identification, which can promote the clinical translation of AI-assisted identification technology. Future study should incorporate multi-center data to construct a multimodal model to further enhance its clinical applicability.

## Data Availability

The original contributions presented in the study are included in the article/**Supplementary Material**. Further inquiries can be directed to the corresponding author.
